# Adipose-derived stem cell exosomes suppress NLRP3-mediated neuronal pyroptosis to attenuate seizures in a kainic acid-induced temporal lobe epilepsy model

**DOI:** 10.3389/fimmu.2025.1691814

**Published:** 2025-10-30

**Authors:** Siqi Ding, Wanying Chen, Yajun E., Jinli Zhou, Songyun Zhao, Yanming Chen, Zhewei Dong, Hao Dai, Yucang He

**Affiliations:** ^1^ The Affiliated Yiwu Hospital of Wenzhou Medical University, Yiwu, Zhejiang, China; ^2^ First Affiliated Hospital of Wenzhou Medical University, Wenzhou, Zhejiang, China

**Keywords:** adipose-derived stem cells, exosomes, temporal lobe epilepsy, NLRP3, pyroptosis

## Abstract

**Background:**

Pyroptosis-mediated neuroinflammation represents a critical pathological mechanism in drug-resistant temporal lobe epilepsy (TLE), while Adipose-derived stem cell exosomes (ADSC-Exos) may target this process through NLRP3 inflammasome inhibition. Our study investigated the therapeutic effects of ADSC-Exos by mitigating NLRP3-driven pyroptosis in TLE.

**Methods:**

We isolated ADSC-Exos, the characteristics of which were confirmed. The Kainic acid-induced mouse TLE model were used to assess the *in vivo* effect of ADSC-Exos. To evaluate ADSC-Exos penetration, brain tissues were collected for fluorescence quantification. TUNEL and Nissl staining were used to evaluate hippocampal neuronal damage. Pyroptosis markers were detected by Western blot, qRT-PCR, and immunofluorescence. Bioinformatics analysis was performed to explore potential miRNAs in ADSC-Exos that might contribute to their therapeutic effects.

**Results:**

Intravenously injected ADSC-Exos efficiently crossed the blood-brain barrier, peaking in brain accumulation at 4 hours post-administration. Treatment with ADSC-Exos resulted in a 48.9% reduction in seizure duration (p<0.0001) and a 42% reduction in spontaneous recurrent seizure frequency (p<0.0001) in temporal lobe epilepsy. Furthermore, ADSC-Exos exhibited significant neuroprotection while suppressing key pyroptosis-related proteins, including NLRP3, Caspase-1, GSDMD, and IL-1β. Bioinformatics analysis further identified 16 candidate miRNAs in ADSC-Exos potentially mediating these therapeutic effects.

**Conclusions:**

ADSC-Exos exert neuroprotective effects in temporal lobe epilepsy in association with regulation of the NLRP3-associated pyroptosis pathway, thereby suppressing neuroinflammation and neuronal death, highlighting their potential therapeutic value.

## Highlights

- ADSC-Exos mitigate neuronal damage and neuroinflammation in temporal lobe epilepsy.
- ADSC-Exos reduce seizure susceptibility and neuronal damage by modulating NLRP3-mediated pyroptosis in temporal lobe epilepsy.
- Bioinformatics reveals 16 neuroprotective miRNAs in ADSC-Exos that may regulate epilepsy-related pathway.

## 1 Introduction

Epilepsy is a chronic brain disorder with diverse etiologies. According to the World Health Organization’s (WHO) 2016 Global Burden of Disease Study, epilepsy ranks as the fourth most burdensome neurological disorder in terms of disability-adjusted life years (DALYs) (1), underscoring its significance as a critical public health concern. Temporal lobe epilepsy (TLE) accounts for 30%-35% of epilepsy cases and is one of the most prevalent yet severe forms of epilepsy, typically characterized by drug resistance, frequent recurrence, and refractory seizures (2, 3). Despite over 20 approved anti-seizure medications (ASMs), approximately 70% of TLE patients develop drug-resistant epilepsy, especially when hippocampal sclerosis is present (4, 5). This highlights the need for novel therapeutic strategies to address the clinical challenges posed by TLE (6).

Inflammation plays a critical role in the pathophysiology of seizures and TLE. Mounting evidence suggests that inflammation could be both a cause and a consequence of epilepsy (7). In epilepsy models, elevated levels of cerebral inflammasomes correlate with neuronal loss and seizure intensity (8). Critically, within this inflammatory cascade, a specific programmed cell death pathway—pyroptosis—has emerged as a key player (9). NLRP1/Caspase-1 signaling has been shown to contribute to neuronal pyroptosis and degeneration in TLE (10). Originally defined by Cookson and Brennan in 2001 (11), pyroptosis is an inflammatory cell death mediated through two pathways: the canonical pathway (via Caspase-1 cleavage) and the non-canonical pathway (via caspase-4/5/11 cleavage) (12). Among various inflammasomes, NLRP3 is the best characterized driver of pyroptosis. Upon sensing cellular stress, NLRP3 assembles with ASC to activate Caspase-1, which cleaves Gasdermin D (GSDMD) and promotes the release of IL-1β/IL-18. This cascade not only executes pyroptotic cell death but also amplifies neuroinflammation. Increasing evidence links aberrant NLRP3 activation with hippocampal neuronal injury and seizure recurrence in temporal lobe epilepsy, highlighting it as a key therapeutic target (13).

Exosomes, a class of extracellular vesicles (EVs) ranging from 30–100 nm in diameter, are endosome-derived nanoparticles that influence intercellular communication by carrying and transferring a diverse array of biomolecules (such as proteins, lipids, and nucleic acids) from one cell to another, thereby modulating the recipient cell’s behavior, gene expression, and signaling pathways (14, 15). Exosome-based therapies offer distinct advantages over stem cell transplantation, including superior biocompatibility, enhanced bioengineering capacity, and reduced risk of abnormal differentiation (14). Adipose-derived stem cell exosomes (ADSC-Exos) exhibit unique properties, such as broader tissue source availability, minimally invasive harvesting, enhanced proliferative potential, and significantly higher exosome yields compared with conventional mesenchymal stem cells (MSCs) (16–18). ADSC-Exos have shown great potential in treating neurological disorders due to these advantages (19, 20).

Our previous studies have identified pyroptosis as a significant pathological mechanism in epileptic mice (21). Although exosome-based strategies have shown neuroprotective effects in stroke and neurodegeneration, whether ADSC-Exos can attenuate temporal lobe epilepsy (TLE) by suppressing NLRP3-mediated pyroptosis remains unknown. This study aims to investigate the anti-pyroptotic effects of ADSC-Exos and their underlying molecular mechanisms in a kainic acid (KA)-induced temporal lobe epilepsy mouse model, with particular focus on the associated signaling transduction pathways.

## 2 Methods

### 2.1 Animals and ethics statement

C57BL/6 mice (6–8 weeks old, male, 20–30 g body weight) were obtained from the Laboratory Animal Center of Wenzhou Medical University. The animals were housed under standard conditions with a 12-hour light/dark cycle and provided with food and water ad libitum. All experimental procedures were approved by the Ethics Committee on Animal Research of the First Affiliated Hospital of Wenzhou Medical University (approval No. WYYY-IACUC-AEC-2025-051) and conducted in strict accordance with the National Institutes of Health Guide for the Care and Use of Animals.

### 2.2 Isolation, cultivation, and induced differentiation of ADSCs

This research was conducted with the approval and supervision of the Clinical Research Ethics Committee at the First Affiliated Hospital of Wenzhou Medical University, assigned the ethical review identifier KY2025-172. Adipose tissue was obtained from donors through standard liposuction procedures performed by qualified clinicians at our institution (22), and then centrifuged at 1200 rpm for 5 minutes. The central layer of adipose tissue was retained, while the other layers were discarded. A volume of Collagenase Type I solution (Sigma, USA, CatNo.9001-12-1) matching that of the adipose tissue was added. A volume of 0.1% Collagenase Type I solution (Sigma, CatNo.9001-12-1) was added to the adipose tissue to achieve a final volume ratio of [1:1]. The mixture was then placed in a shaker set to 37°C and agitated for 60 minutes. To stop the enzymatic process, an equivalent volume of serum-supplemented medium (containing 10% FBS) was added to the solution to neutralize the enzymatic digestion. The digested tissue was filtered using a 70-μm mesh to produce a single-cell suspension. This suspension was centrifuged at 1000 rpm for 5 minutes, after which the upper liquid was removed, and the remaining cell pellet was resuspended in a suitable growth medium. The isolated cells were transferred to culture dishes and incubated at 37°C in a humidified environment containing 5% CO2. To promote cell growth and passaging, the culture medium (Oricell, China, CatNo.HUXMD-90012) was replaced every three days. To characterize the properties of ADSCs, third passage cells were cultured and induced to differentiate, and their adipogenic, osteogenic, and chondrogenic differentiation capabilities were validated using Oil Red O (Oricell, CatNo. OILR-10001), Alizarin Red S (Oricell, CatNo. ALIR-10001), and Toluidine Blue O staining (MCE, CatNo.HY-D0220), respectively. Surface marker characterization of ADSCs was performed by flow cytometry using a BD FACSAria™ system (BD Biosciences, NJ, USA). Third-passage ADSCs were resuspended at a density of 1×10^6^ cells/mL and stained with Phycoerythrin-conjugated antibodies against CD29 (BioLegend, USA, CatNo.303001), CD31 (BioLegend, CatNo.375902), CD44 (BioLegend, CatNo. 338802), CD45 (Multi Sciences, China, CatNo.F11045A02), and CD90 (BioLegend, CatNo.389802) for 30 min at 4°C. After incubation, cells were washed and analyzed. PE-labeled mouse IgG (Multi Sciences, China, CatNo.F11IG102) served as the isotype control. Following incubation, cells were washed twice with PBS containing 1% bovine serum albumin, centrifuged at 1000rpm for 5 minutes, and then resuspended in PBS for analysis. Data were acquired on the BD FACSAria™ system and analyzed using FlowJo software (v10, BD Life Sciences).

### 2.3 Extraction and identification of ADSC-Exos

ADSCs from passages 3 to 5 were cultivated in a complete medium free of exosomes for at least 48 hours. The liquid above the cells was then collected for further processing. Exosomes were separated using a density gradient ultracentrifugation technique. The collected liquid underwent a series of centrifugation steps at 300g, 2000g, and 10000g, each lasting 30 minutes. After each step, the sediment was discarded, and the remaining liquid was used for the next centrifugation. The resulting liquid was then subjected to ultracentrifugation at 120000g for 70 minutes, repeated twice. After each round, the supernatant was removed, and the pellet was kept. The exosome-containing pellet was dissolved in phosphate-buffered saline (PBS), and stored at -80°C for subsequent analysis.

The characteristic morphology of ADSC-Exos was captured by transmission electron microscopy (TEM; JEOL, Japan). Particle size distribution profiles were quantitatively assessed through nanoparticle tracking analysis (NTA) using a Malvern Instruments system. Total protein levels of isolated exosomes were measured with a bicinchoninic acid (BCA) assay kit (Beyotime, China, CatNo.P0010). Exosomal identity was further verified via Western blot detection of specific markers: CD9 (Affinity, AF5139; 1:1000), CD63 (Affinity, AF5117; 1:1000), and TSG101 (Affinity, DF8427; 1:1000).

### 2.4 PKH26 labeling to depict ADSC-Exos in brain parenchyma

According to the instructions of the PKH26 Red Fluorescent Cell Linker Mini Kits (Sigma-Aldrich,CatNo.PKH26PCL) for labeling exosomes, the PKH26-labeled exosomes were resuspended in PBS buffer and injected via the tail vein into three normal mice (100μg, 0.2 ml each). After 2 hours, 4 hours, and 6 hours, the mice were deeply anesthetized, followed by cardiac perfusion to collect the whole brain tissue for the preparation of frozen tissues and sections. The sections were fixed in pre-cooled anhydrous methanol for 15 minutes, followed by washing with Tris-buffered saline with Tween buffer three times for 5 minutes each to remove the anhydrous methanol. Under dark conditions, Hoechst staining solution was added to stain the nuclei, and the PKH26 red fluorescent-labeled exosomes were observed under a fluorescence microscope.

### 2.5 TLE models and grouping

The mouse TLE model was established by intrahippocampal injection of KA (0.5 μg, 0.5 μl) using a stereotactic apparatus. Seizure severity was assessed using Racine’s scale (23), which is detailed as follows: Stage 0, no response; Stage 1, ear and facial twitching; Stage 2, myoclonic jerks (MJs); Stage 3, clonic forelimb convulsions; Stage 4, generalized clonic seizures with turning to a side position; and Stage 5, generalized tonic–clonic seizures (GTCSs) or death. Only seizures with a Racine score of 4–5 were considered substantial to induce subsequent spontaneous seizures.

**Figure 1:**
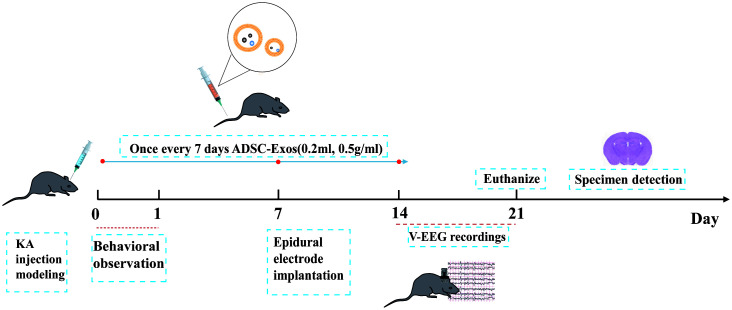
Schematic illustration of experimental setup and timeline. After KA-induced epilepsy modeling in mice, seizure severity was assessed via 24-hour behavioral observation. On day 7 post-modeling, epidural electrodes were implanted with a 7-day recovery period. Continuous 7-day EEG-video monitoring was performed from days 14–21. Adipose-derived stem cell exosomes were administered intravenously on days 1, 7, and 14. On day 21, mice were euthanized for sample collection.

Mice were randomly divided into four groups, with a target of five surviving animals per group. Based on prior reports of ~20% mortality during KA-induced status epilepticus, six mice were initially enrolled in the KA group. As expected, one KA mouse died during the modeling process, leaving five animals for final analysis. Thus, the final group sizes were Control (n = 5), KA-ADSC-Exos (n = 5), KA-Nig-ADSC-Exos (n = 5), and KA (n = 5). Group A (Control) underwent sham surgery with hippocampal injection of 0.5 μl normal saline, followed by tail vein administration of 0.2ml normal saline; Group B (KA-ADSC-Exos) received hippocampal KA injection followed by tail vein administration of ADSC-Exos solution (0.2ml,0.5mg/ml); Group C (KA-Nig-ADSC-Exos) received KA injection, followed by NLRP3 activator Nigericin (4 mg/kg, i.p.), and then tail vein ADSC-Exos solution(0.2ml,0.5mg/ml); Group D (KA) received hippocampal KA injection to induce temporal lobe epilepsy followed by tail vein saline (0.2 mL); The ADSC-Exos injections were administered three times in total, on day 1, day 7, and day 14. Behavioral monitoring was conducted for one day following the first injection. After the final injection on day 14, continuous behavioral observation was carried out for seven days, and the subjects were euthanized on day 21 (**Figure 1**).

### 2.6 Observation of epileptic seizure

Twenty-four hours after KA injection, seizure severity was evaluated through direct behavioral observation for 24 consecutive hours. Seizures were scored according to the modified Racine scale (stage 4 or higher), and the average seizure score was calculated to quantify epileptic severity.

After two weeks of KA administration, video electroencephalogram (EEG) recordings were performed to monitor seizures in KA-treated mice. One week after KA modeling, an epidural electrode implantation surgery was conducted under isoflurane anesthesia, with the mice positioned in a stereotaxic frame. Screws were implanted on both sides of the skull near the temporal lobe (AP: -3.2 mm; ML: ± 2.9 mm) for recording positive and negative electrodes, and a screw was placed in the frontal bone as a ground electrode. All screws were soldered to a custom electrode for the mice and secured with dental cement. Mice were given a recovery period of seven days post-surgery before EEG recordings commenced. A digital video-EEG acquisition system was used for continuous monitoring of the mice for seven days, along with 24-hour synchronized video recording of the EEG. Following the recording, statistical analyses of all EEG data for each monitoring period were conducted, and videos were analyzed as needed to confirm the behavioral correlation of seizure activities. The average duration of spontaneous recurrent seizures (SRSs) and the number of SRSs in mice were statistically evaluated. Spontaneous seizures were characterized by evolving spike-wave discharges with a frequency >2 Hz, an amplitude at least 3 times greater than baseline, and a duration of ≥15 seconds (24).

### 2.7 Western blotting

Protein levels were assessed using immunoblotting. All mice were administered 1% pentobarbital sodium and subsequently decapitated, allowing for the immediate collection of the bilateral hippocampus. Hippocampal tissue samples (20–30 mg) were lysed, and the resulting mixtures were centrifuged at 12,000 g for 15 minutes to obtain the supernatants. A total of 20 μg of protein samples were then separated using Sodium Dodecyl Sulfate-Polyacrylamide Gel Electrophoresis (10% separation gel) and transferred onto a Polyvinylidene Fluoride membrane (Merck and Co., Inc., Whitehouse Station, NJ, USA, CatNo.03010040001). The Polyvinylidene Fluoride membranes were blocked with 5% nonfat milk at room temperature for 1 hour. Following this, the membranes were incubated overnight at 4°C with the following primary antibodies: NLRP3 (BA3677, 1:1,000; Boster Biological Technology), GSDMD (AF4012, 1:1,000; Affinity), Caspase-1 (ET1068-69, 1:3,000; Hua An Biotechnology), IL-1β (bs-6319R, 1:1,000; Bioss), and β-actin (AC026, 1:50,000; Abclonal). After washing the membranes three times, HRP-conjugated goat anti-rabbit (111-035-003, 1:5,000; Jackson) and goat anti-mouse IgG (SA00001-1, 1:5,000; San Ying Biotechnology, Wuhan, China) secondary antibodies were applied. The membranes were incubated for 30 minutes at room temperature. Protein detection was conducted using the ECL (Enhanced Chemiluminescence) method (Bio-Rad,USA,CatNo.1705062), and the protein levels were normalized to β-actin as an internal control.

### 2.8 qRT-PCR

mRNA expression in the hippocampus was assessed through real-time quantitative qRT-PCR. Total RNA was isolated using TRIzol reagent (Thermo Fisher Scientific, China, CatNo.15596018CN). The RNA concentration was determined with a NanoDrop One spectrophotometer. Following the manufacturer’s instructions for the reverse transcription kit (Sigma, USA, CatNo.KCQS02), a reverse transcription reaction was set up, allowing 500 ng of RNA to be converted into cDNA. This synthesized cDNA was then subjected to qRT-PCR using SYBR (SYBR Green Supermix). All results were normalized to β-actin. All qRT-PCR primer sequences are provided in **Supplementary Table S1**.

### 2.9 ELISA

IL-1β and IL-18 levels in mouse serum were measured using ELISA kits (R&D Systems, CatNo: DY401,DY122-05) following the manufacturer’s protocol. Blood was collected via retro-orbital puncture, allowed to clot at room temperature for 30 min, and centrifuged (2000×g, 10 min) to obtain serum. Standards and samples were added to pre-coated wells, incubated for 2 h, and processed with detection antibodies (1 h), streptavidin-HRP (30 min), and substrate solution (20 min). Absorbance (450 nm) was measured, and concentrations were calculated from the standard curve. All the procedures were repeated for at least three times.

### 2.10 Nissl staining and TUNEL staining

The process involves deep anesthesia and cardiac perfusion of the mouse, followed by fixation of the brain tissue in 4% paraformaldehyde, dehydration with sucrose solutions, and embedding in Optimal Cutting Temperature compound for rapid freezing. After obtaining 20-micron sections using a cryostat, the sections are stained using the Nissl staining method, then washed and mounted on slides for microscopic observation and analysis.

To evaluate pyroptotic cell death, tissue cryosections were subjected to TUNEL (TdT-mediated dUTP Nick-End Labeling) staining using fluorescein-dUTP to label DNA fragmentation in apoptotic nuclei, while DAPI (4′,6-diamidino-2-phenylindole; 1 μg/mL) was used to counterstain all nuclei. The assay was performed according to the manufacturer’s protocol (TUNEL Apoptosis Detection Kit, R&D Systems, Switzerland,CatNo.4810-30-CK).

### 2.11 GEO dataset collection

To investigate the miRNA expression profiles associated with epilepsy and adipose-derived stem cell exosomes, we retrieved two publicly available datasets from the GEO (Gene Expression Omnibus) website (http://www.ncbi.nlm.nih.gov/geo/). The GSE99455 (25, 26) dataset included hippocampal miRNA profiling of 16 patients with medically intractable epilepsy and 8 postmortem healthy controls. The raw fastq files and processed miRNA expression matrices were downloaded for subsequent bioinformatic analysis. To analyze the non-coding RNA profile of adipose-derived mesenchymal stem cell (ADMSC)-derived exosomes, we used dataset GSE281527 (27) from GEO, selecting three exosome samples (ADMSCs-Exo) analyzed by microarray (Agilent miRNA platform). Both datasets were subjected to rigorous quality control procedures.

### 2.12 Statistical analysis

Statistical analyses were conducted using GraphPad Prism 7 (GraphPad, San Diego, CA, United States). The quantitative analysis of Western Blot was performed using ImageJ software (version 1.54). The difference in severe seizure incidence between groups was assessed using the chi-square test (χ²). For normally distributed continuous data: Two-group comparisons were made using Student’s t-test (mean ± SD). One-way or two-way ANOVA with Tukey’s *post hoc* tests when appropriate. Non-normally distributed variables were analyzed with the Mann-Whitney U test and expressed as median (IQR). Statistical significance was set at p < 0.05.

## 3 Results

### 3.1 Characterization of ADSCs and ADSC-Exos

**Figure 2:**
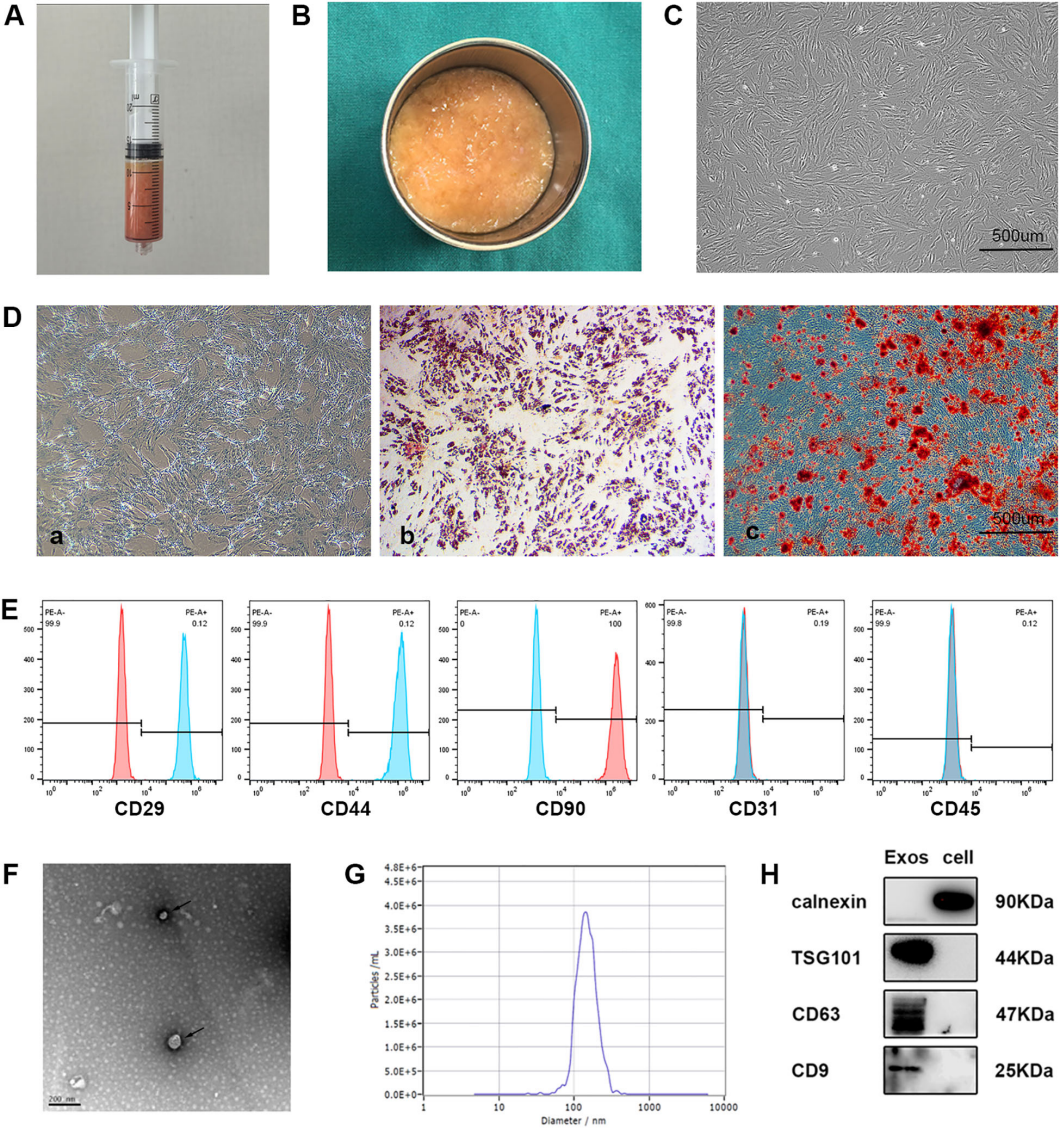
Characterization of ADSCs and exosomes. **(A)** Collection of adipose tissue obtained immediately by liposuction. **(B)** Adipose tissue after oil and water removal. **(C)** Representative image showing the typical spindle-like shape of isolated ADSCs under a light microscope. **(D)** Representative images of ADSCs differentiated into chondrocytes, osteogenic cells, and adipocytes, stained with Alcian Blue **(a)**, Alizarin Red S **(b)**, and Oil Red O **(c)**, respectively. **(E)** Flow cytometry showed that CD29, CD44 and CD90 was positive and CD31, or CD45 was negative among the surface markers ADSCs. **(F)** Transmission electron microscopy (TEM) image of ADSCs derived exosomes revealing cup-shaped morphology (black arrows). **(G)** Nanoparticle tracking analysis (NTA) showing the size distribution of ADSCs derived exosomes. **(H)** Western blot analysis of CD63, TSG101, CD9, calnexin in ADSC-Exos and ADSCs (n = 3 per group).

Adipose tissue acquired through liposuction was immediately processed via gravitational sedimentation to remove aqueous and lipid phases (**Figures 2A, B**). Primary ADSCs isolated from this tissue demonstrated characteristic spindle-shaped morphology under phase-contrast microscopy (**Figure 2C**). Multilineage differentiation assays verified trilineage potential through chondrogenic (Alcian blue staining), osteogenic (Alizarin red staining), and adipogenic differentiation (Oil Red O staining) capacities (**Figures 2Da–c**). Flow cytometric profiling further established constitutive expression of mesenchymal markers (CD29/CD44/CD90) with absence of hematopoietic (CD45) and endothelial (CD31) markers, fully compliant with International Society for Cellular Therapy (ISCT) criteria [27] (**Figure 2E**). Subsequent ultracentrifugation-based isolation of exosomes (ADSC-Exos) from conditioned medium yielded three distinct validations: cup-shaped nanostructures observed by TEM (**Figure 2F**); a monodisperse size distribution peaking at 142.9 nm via NTA (**Figure 2G**); and biochemical confirmation of enriched exosomal markers (CD63/TSG101/CD9) coupled with undetectable endoplasmic reticulum contaminant calnexin in Western blots (**Figure 2H**), collectively substantiating exosomal purity.

### 3.2 ADSC-Exos crossed the blood-brain barrier

**Figure 3:**
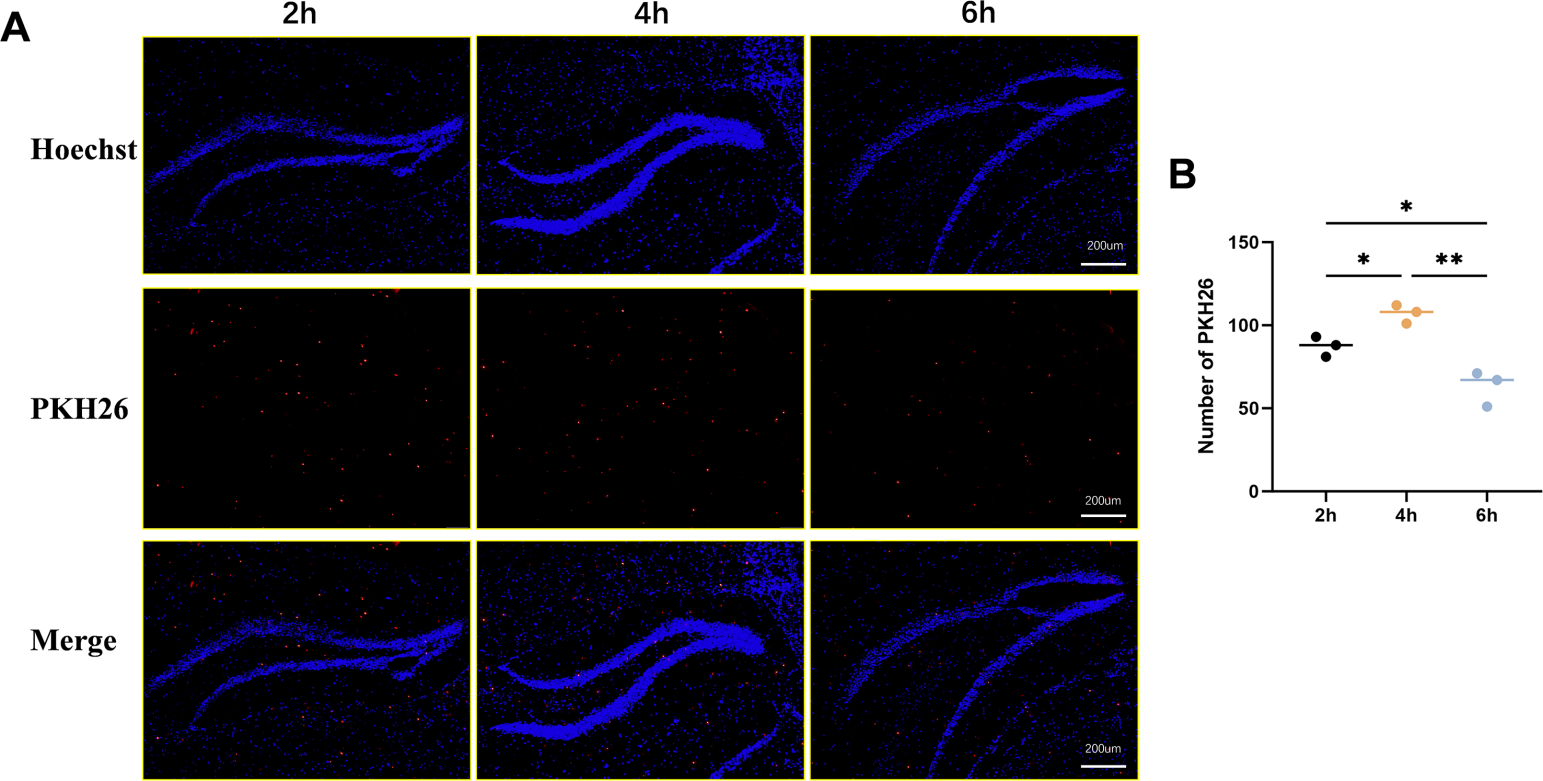
PKH26 labeling to depict ADSC-Exos in brain parenchyma. **(A)** Representative image shows that PKH26-labeled ADSC-Exos cross the blood-brain barrier and accumulate in brain tissue over time. Fluorescence images show PKH26-labeled exosomes (red) in brain tissue at 2, 4, and 6 h post-injection (left to right). Nuclei were stained with Hoechst (blue). Strongest exosome accumulation was observed at 4 (h) **(B)** Quantitative analysis of the relative expression of PKH26-labeled ADSC-Exos in Hippocampus. Individual data points represent values from each mouse (n=3 per group). Data are expressed as mean ± SD. *p < 0.05, **p < 0.01(one-way ANOVA with Tukey’s *post hoc* test).

To investigate the ability of exosomes to cross the blood-brain barrier (BBB), PKH26-labeled ADSC-Exos were administered via tail vein injection. Brain tissues were collected at 2, 4, and 6 hours post-administration, and frozen sections were prepared for immunofluorescence microscopy. As shown in **Figure 3A**, red fluorescence signals corresponding to PKH26-labeled exosomes were observed in brain tissue, predominantly clustered around blue-stained nuclei, confirming successful penetration of exosomes across the BBB into the brain parenchyma. Notably, the red fluorescence intensity was most pronounced at 4 hours post-administration, indicating peak exosome accumulation in the brain tissue at this time point, with relatively weaker signals observed at 2 and 6 hours (**Figure 3B**). These findings demonstrate that exosomes can effectively cross the BBB and accumulate in brain tissue, with optimal penetration observed at 4 hours post-administration.

### 3.3 ADSC-Exos significantly attenuated seizures in KA-induced TLE mice

**Figure 4:**
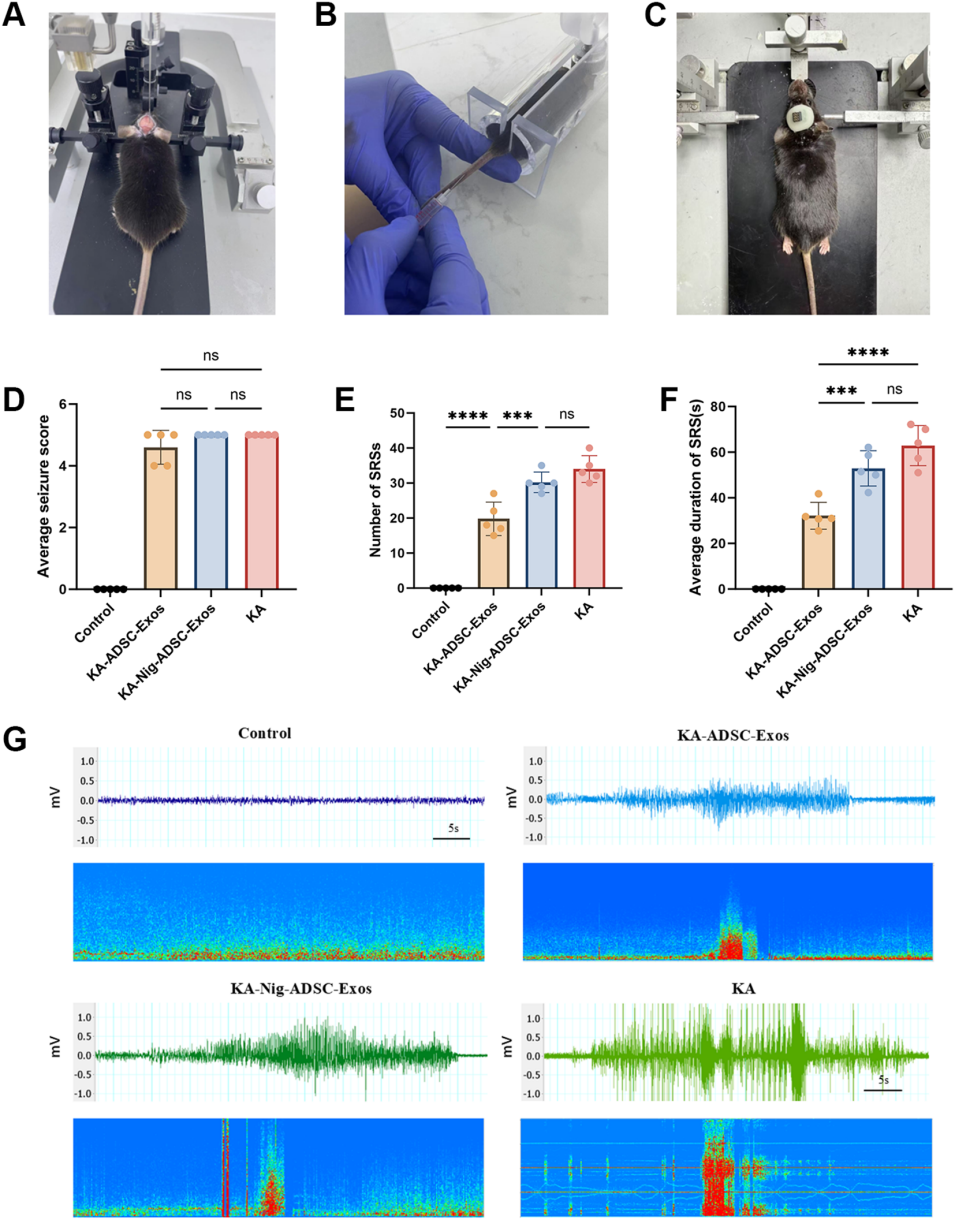
Therapeutic effects of ADSC-Exos on seizure activity in KA-induced temporal lobe epilepsy (TLE) mice. **(A)** Stereotaxic intrahippocampal KA injection (coordinates: AP −2.0 mm, ML −1.8 mm [right hemisphere], DV −1.8 mm). **(B)** Intravenous administration of ADSC-Exos via tail vein. **(C)** Surgical implantation of epidural EEG electrodes. **(D–F)** Quantitative analyses of **(D)** average seizure scores on Day 1 post-KA injection, **(E)** frequency of SRSs during the chronic phase (days 14–21), and **(F)** mean duration of spontaneous recurrent seizures (SRSs). Individual data points represent values from each mouse (n=5 per group). Data are expressed as mean ± SD. Statistical significance: *p < 0.05, **p < 0.01, ***p < 0.001, ****p < 0.0001(one-way ANOVA with Tukey’s *post hoc* test). **(G)** Representative 1-minute EEG traces during epileptic episodes.

**Figure 4A** illustrates KA epilepsy modeling, **Figure 4B** details exosome intravenous injection, and **Figure 4C** depicts epidural electrode implantation. After modeling, during the acute phase (day 1 post-KA), the Control group exhibited no seizures, while no significant differences in average seizure stage scores were observed among KA, KA-ADSC-Exos, and KA-Nigericin-ADSC-Exos groups (**Figure 4D**). In the chronic phase (days 14–21), the Control group remained seizure-free, while the KA group demonstrated the highest spontaneous recurrent seizure (SRS) frequency and longest average SRS duration. Compared to the KA group, the KA-ADSC-Exos group exhibited significant reductions in both SRS frequency and duration (**Figures 4E, F**), indicating optimal anti-seizure efficacy. Although the KA-Nigericin-ADSC-Exos group showed moderate reductions in SRS metrics versus the KA group (**Figures 4E, F**), its values remained significantly elevated relative to the KA-ADSC-Exos group. Representative seizure recordings for each group are shown in **Figure 4G**. These results indicate that ADSC-Exos robustly attenuate chronic epileptogenesis while Nigericin-induced pyroptosis partially diminishes their therapeutic effect, though KA-ADSC-Exos maintains superior anti-epileptic potential.

### 3.4 Neuroprotective effects of ADSC-Exos on KA-induced hippocampal damage

**Figure 5:**
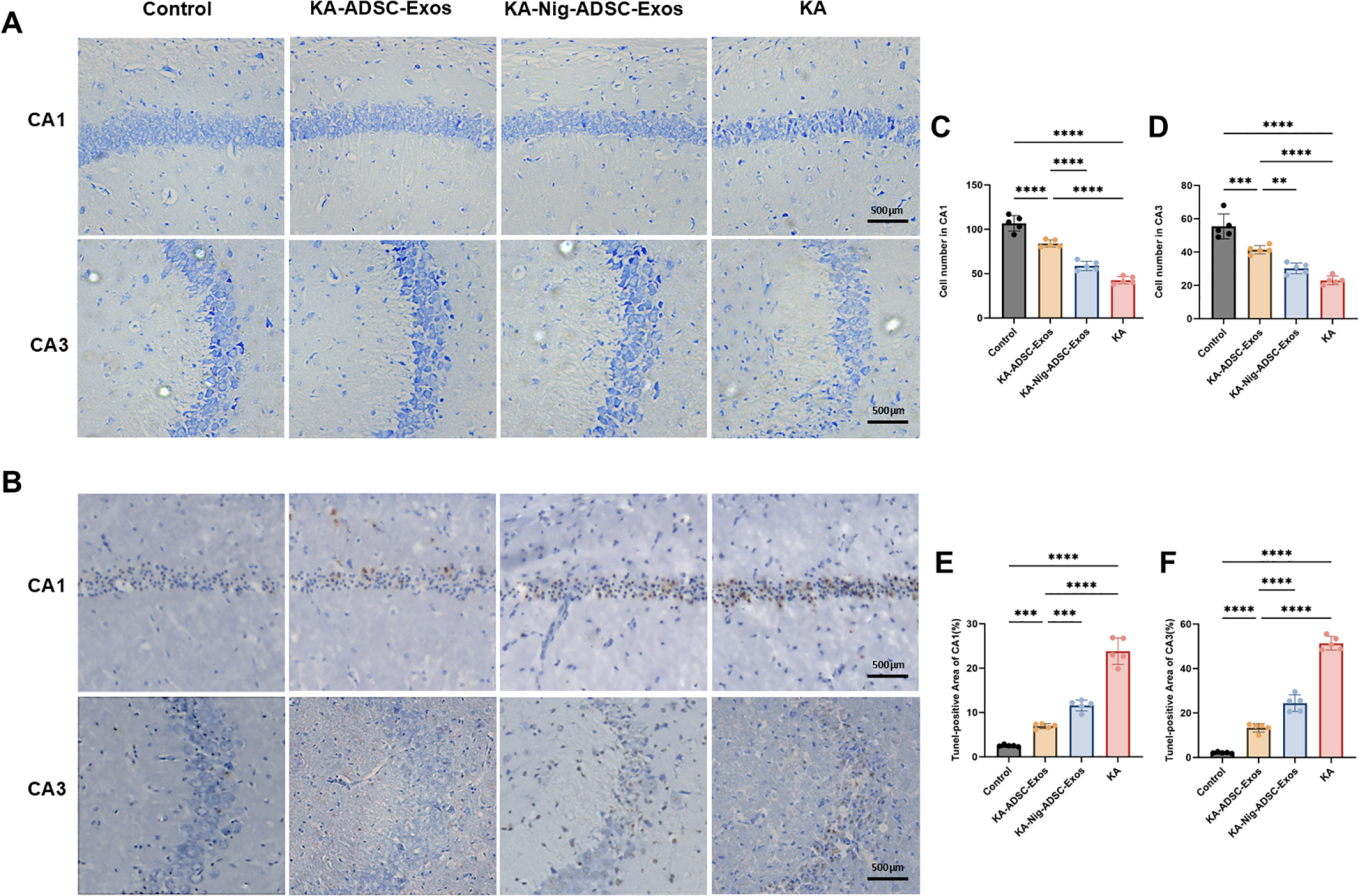
Effects of ADSC-Exos on KA-induced hippocampal neuron damage. **(A)** Representative Nissl-stained images (200×) showing ADSC-Exos attenuated KA-induced neuron loss in hippocampal CA1 and CA3 regions. **(B)** Representative TUNEL-stained images (200×) showing ADSC-Exos alleviated KA-induced apoptosis in CA1 and CA3. **(C–F)** Quantitative analyses of **(C)** CA1 Nissl+, **(D)** CA3 Nissl+, **(E)** CA1 TUNEL+, and **(F)** CA3 TUNEL+ cells. Individual data points represent values from each mouse (n=5 per group). Data are expressed as mean ± SD. *p < 0.05, **p < 0.01, ***p < 0.001, ****p < 0.0001 (one-way ANOVA with Tukey’s *post hoc* test).

We examined the effects of ADSC-Exos on hippocampal neuronal damage induced by KA injection (**Figure 5**). Nissl staining (**Figure 5A**) and TUNEL staining (**Figure 5B**) were performed on hippocampal from four groups. As shown in **Figure 5**, hippocampal CA1 and CA3 areas of mice in the KA group showed serious damage compared with those of the control group. The KA-ADSC-Exos group showed significant neuroprotection, with ADSC-Exos effectively mitigating KA-induced hippocampal neuronal damage and markedly increasing neuronal survival rates in both CA1 and CA3. The KA-Nigericin-ADSC-Exos group demonstrated moderate protection compared to the KA group, evidenced by increased neuronal density and decreased Tunel-positive cells in CA1 and CA3, though its effects were less pronounced than those of the KA-ADSC-Exos group. Additionally, histological examination revealed that ADSC-Exos exerted a more prominent neuroprotective effect in the CA1 region compared to CA3.

### 3.5 ADSC-Exos suppress NLRP3 inflammasome activation and pro-inflammatory cytokines in KA-induced TLE mice

**Figure 6:**
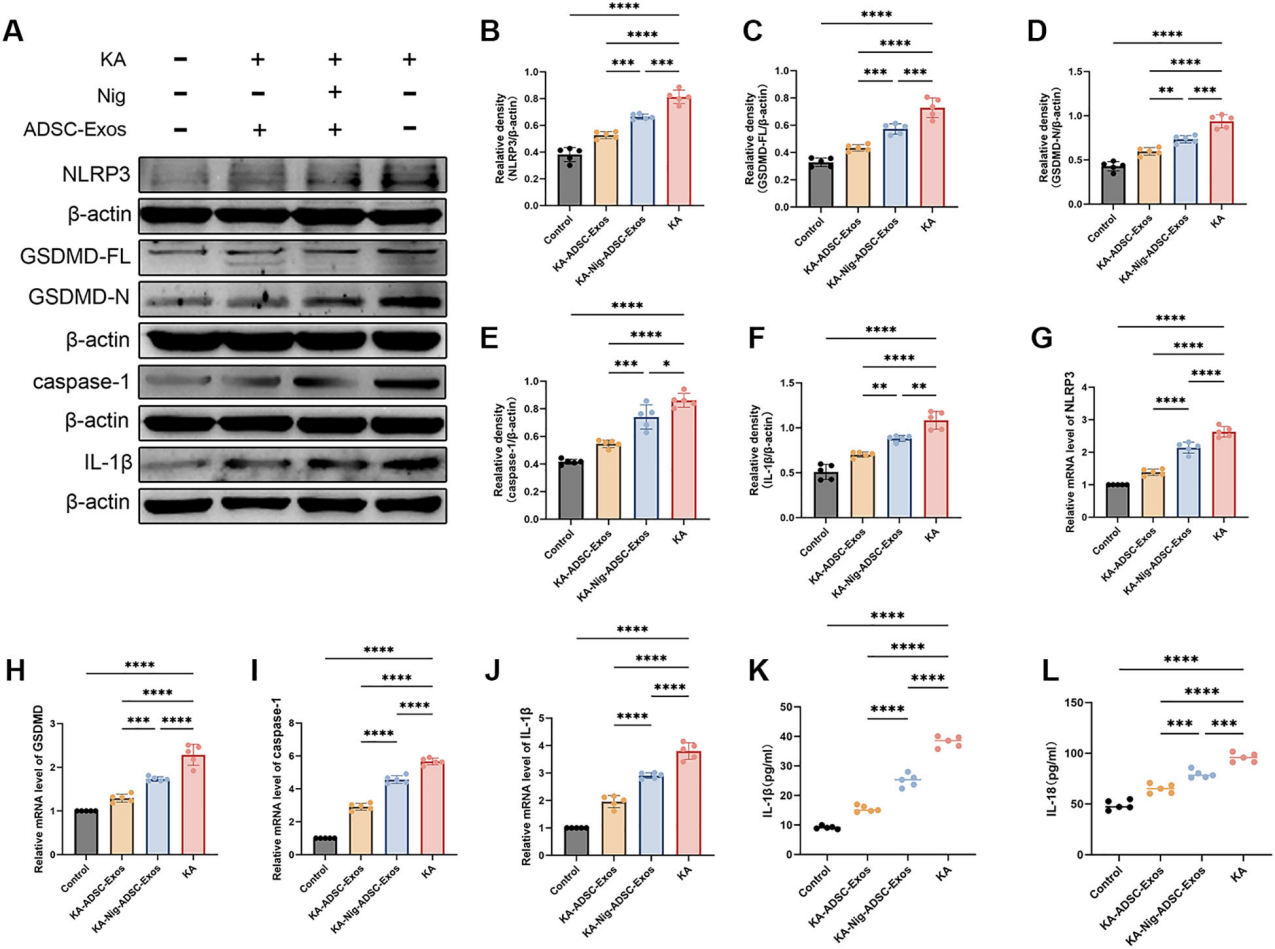
ADSC-Exos mitigate NLRP3-mediated pyroptosis in KA-induced temporal lobe epilepsy. **(A–F)** Western blot analysis and quantification of **(A)** representative immunoblots and protein levels of **(B)** NLRP3, **(C)** GSDMD-FL, **(D)** GSDMD-N, **(E)** Caspase-1, and **(F)** IL-1β in the Control, KA, KA+ADSC-Exos, and KA+Nig+ADSC-Exos groups. **(G–J)** qRT-PCR analysis of relative mRNA expression of **(G)** NLRP3, **(H)** GSDMD, **(I)** Caspase-1, and **(J)** IL-1β. **(K–L)** ELISA quantification of **(K)** serum IL-1β and **(L)** IL-18 levels. Individual data points represent values from each mouse (n=5 per group). Data are expressed as mean ± SD. *p < 0.05, **p < 0.01, ***p < 0.001, ****p < 0.0001 (one-way ANOVA with Tukey’s *post hoc* test).

Given the critical role of neuroinflammation in epilepsy and neuronal damage, we further investigated the involvement of the NLRP3 inflammasome, a key mediator of inflammatory responses. To elucidate the effects of KA-induced kindling on NLRP3 inflammasome activation, we assessed the expression levels of NLRP3 and its associated components in the hippocampus at both protein and mRNA levels. As shown in **Figure 6**, the KA group exhibited a marked upregulation of NLRP3 protein and mRNA expression compared to the control group, indicative of robust inflammasome activation. Additionally, The KA group showed significantly increased expression of NLRP3, Caspase-1, GSDMD, and IL-1β, further amplifying the inflammatory cascade. Remarkably, ADSC-Exos treatment significantly suppressed KA-induced NLRP3 inflammasome activation. In the KA-ADSC-Exos group, both NLRP3 protein and mRNA levels were substantially reduced, accompanied by decreased expression of Caspase-1, GSDMD and IL-1β. To validate these findings, ELISA was employed to measure the levels of pro-inflammatory cytokines IL-1β and IL-18 in hippocampal tissue. Consistent with the Western blot results, ELISA analysis confirmed a significant reduction in IL-1β and IL-18 levels in the ADSC-Exos-treated group compared to the KA group. These findings underscore the potent anti-inflammatory properties of ADSC-Exos, which likely contribute to their neuroprotective effects by attenuating NLRP3 inflammasome-driven neuroinflammation.

### 3.6 Identification of ADSC-Exos-derived miRNAs targeting NLRP3 inflammasome pathways in epilepsy

**Figure 7:**
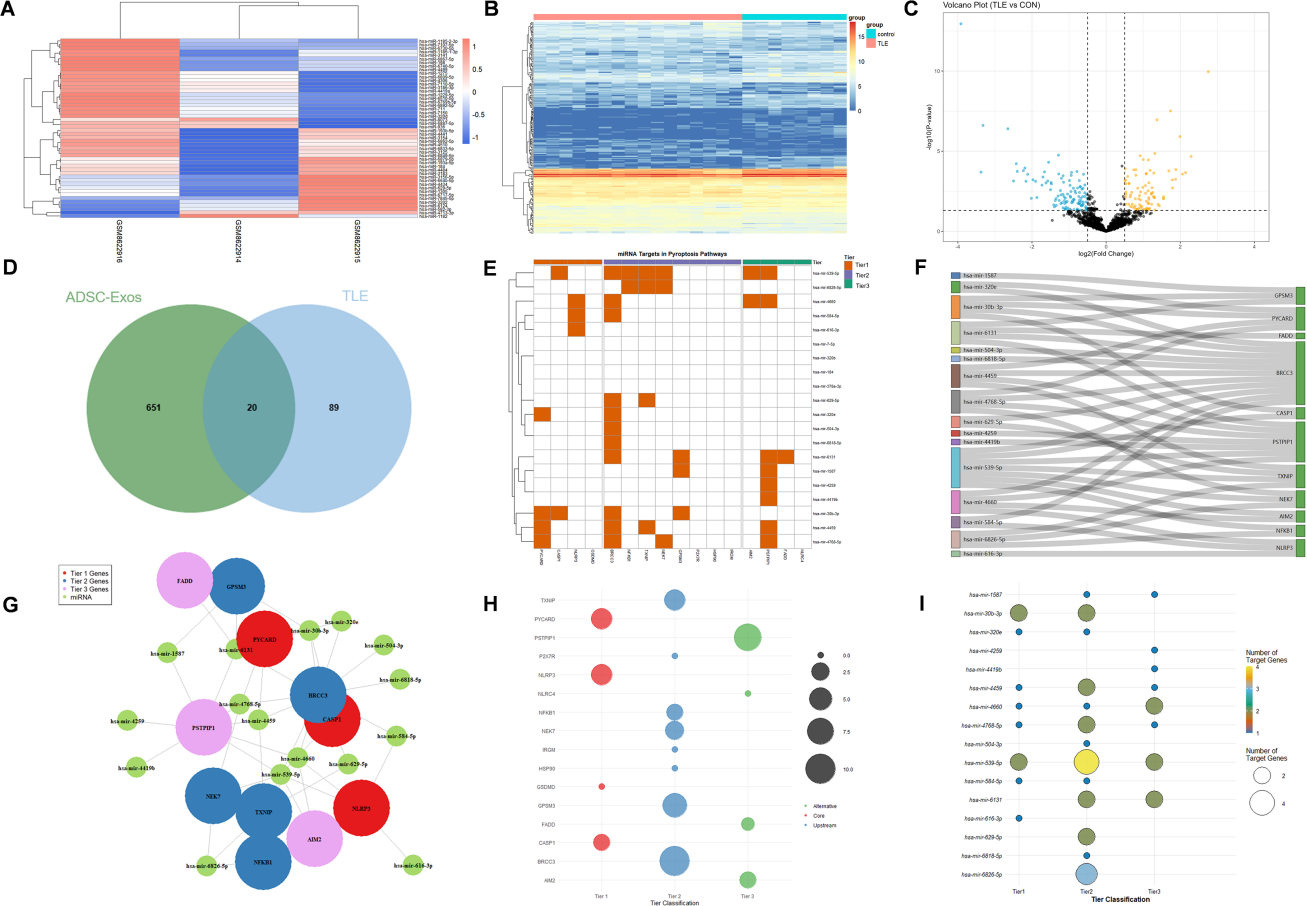
Bioinformatics analysis of hippocampal miRNA profiles from patients with refractory epilepsy (GSE99455, GEO database) and ADSC-derived exosomal miRNAs (GSE281527, GEO database). **(A)** Heatmap showing the top 50 most abundant miRNAs in ADSC-Exos. **(B)** Heatmap of 199 significantly downregulated hippocampal miRNAs (|log2FC| > 1, adj. p < 0.05) in epileptic patients versus healthy controls. **(C)** Volcano plot of differentially expressed miRNAs in the epileptic hippocampus. **(D)** Venn diagram showing 20 shared miRNAs that were enriched in ADSC-Exos and downregulated in epileptic hippocampi. **(E–I)** miRNA–target interaction analyses of 20 key miRNAs (hsa-mir-1587, hsa-mir-184, hsa-mir-30b-3p, hsa-mir-320b, hsa-mir-320e, hsa-mir-378a-3p, hsa-mir-4259, hsa-mir-4419b, hsa-mir-4459, hsa-mir-4660, hsa-mir-4768-5p, hsa-mir-504-3p, hsa-mir-539-5p, hsa-mir-584-5p, hsa-mir-6131, hsa-mir-616-3p, hsa-mir-629-5p, hsa-mir-6818-5p, hsa-mir-6826-5p, and hsa-mir-7-5p): **(E)** Heatmap showing differential expression of key miRNA–target pairs. **(F)** Sankey diagram illustrating miRNA–gene–pathway relationships. **(G)** Network visualization of core miRNA–target interactions. **(H)** Bubble plot ranked by target genes (y-axis: enriched terms). **(I)** Bubble plot ranked by regulating miRNAs (y-axis: miRNA clusters).

To preliminarily explore the molecular mechanisms underlying the potential anti-epileptic effects of ADSC-Exos, we conducted a bioinformatics analysis of hippocampal miRNA profiles from patients with intractable epilepsy (GSE99455) alongside the miRNA signatures of ADSC-Exos (GSE281527). **Figure 7A** displays the top 50 most highly abundant miRNAs in ADSC-Exos from the GSE281527 dataset. Differential expression analysis of hippocampal miRNA profiles from medically intractable epilepsy patients compared to healthy controls (GSE99455) identified 199 significantly downregulated miRNAs (|log2FC| > 1, adj. p < 0.05), as shown in **Figure 7B** (heatmap) and **Figure 7C** (volcano plot). Intersection analysis of these datasets (Venn diagram, **Figure 7D**) identified 20 overlapping miRNAs that were both highly abundant in ADSC-Exos and downregulated in epileptic hippocampi: hsa-mir-1587, hsa-mir-184, hsa-mir-30b-3p, hsa-mir-320b, hsa-mir-320e, hsa-mir-378a-3p, hsa-mir-4259, hsa-mir-4419b, hsa-mir-4459, hsa-mir-4660, hsa-mir-4768-5p, hsa-mir-504-3p, hsa-mir-539-5p, hsa-mir-584-5p, hsa-mir-6131, hsa-mir-616-3p, hsa-mir-629-5p, hsa-mir-6818-5p, hsa-mir-6826-5p, and hsa-mir-7-5p. Through KEGG pathway analysis and literature review, we preliminarily identified three tiers of components in the NLRP3–pyroptosis pathway: (1) core effectors (NLRP3, PYCARD, CASP1, GSDMD); (2) upstream regulators (NFKB1, TXNIP, NEK7, P2X7R, HSP90, IRGM, GPSM3, BRCC3); and (3) alternative inflammasome components (AIM2, NLRC4, PSTPIP1, FADD). TargetScan analysis indicated that the 20 candidate miRNAs putatively target genes across this axis, and 16 of these miRNAs were prioritized as regulators of key components. Correspondingly, the heatmap (**Figure 7E**), Sankey diagram (**Figure 7f**), network view (**Figure 7G**), and bubble plots (**Figures 7H–I**) together depict the resulting miRNA–target relationships.

## 4 Discussion

**Figure 8:**
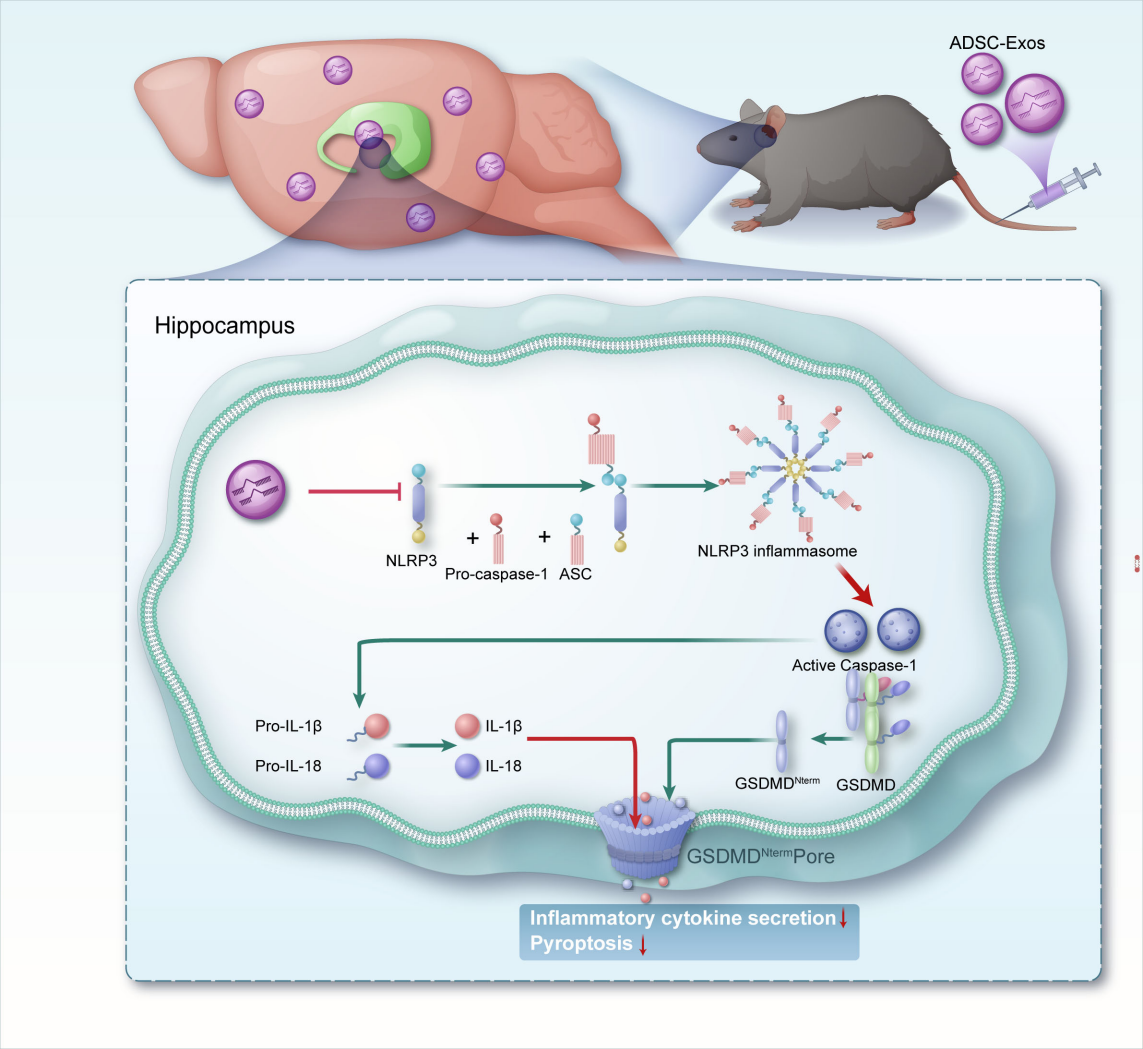
A schematic representation illustrates mechanisms by which adipose-derived stem cell exosomes suppress NLRP3-mediated pyroptosis to alleviate temporal lobe epilepsy.

In the KA-induced TLE model, we provide the first evidence that the anti-epileptic effects of ADSC-Exos are associated with the suppression of NLRP3 inflammasome-mediated pyroptosis and with seizure attenuation (**Figure 8**), a mechanism distinct from previous reports focusing on microglial modulation or oxidative stress reduction by ADSC-Exos (28, 29). Behavioral analyses revealed reduction in seizure severity and frequency following ADSC-Exos treatment. Additionally, histological and molecular analyses showed diminished neuronal loss and neuroinflammation in the hippocampus. Mechanistically, ADSC-Exos suppressed the activity of the NLRP3 inflammasome, leading to reduced Caspase-1 activation and lower IL-1β release, which in turn mitigated pyroptotic cell death. These findings suggest that ADSC-Exos offer neuroprotective effects in TLE, by modulating NLRP3-dependent pyroptosis, pointing to a promising therapeutic approach targeting neuroinflammatory mechanisms in epilepsy.

Stem cell-mediated therapy represents a promising approach for treating neurological diseases (30). Among various stem cells, ADSCs have emerged as a high-quality source of MSCs due to their ease of acquisition and high abundance (31), offering greater potential for scalable applications compared to MSCs derived from bone marrow or umbilical cord (32, 33). The primary therapeutic effect of ADSCs arises from their paracrine functions rather than direct differentiation into neural cells (34). Notably, exosomes serve as key mediators, inheriting the therapeutic potential of stem cells while providing distinct delivery advantages. Leveraging their small size and natural membrane structure, these exosomes can efficiently penetrate the blood-brain barrier and evade immune clearance, enabling the precise delivery of functional miRNAs and proteins (35).

This study utilized PKH26 fluorescent labeling and tail vein injection of ADSC-Exos to assess their distribution within the brain tissue of normal mice. Experimental results demonstrated that ADSC-Exos crossed the blood-brain barrier at the 2nd, 4th, and 6th hour post-injection, achieving widespread distribution in hippocampal and cortical regions-a finding consistent with prior research (36, 37). The fluorescent signal was most pronounced at the 4th hour. Although seizure frequency and neuronal damage were evaluated during later chronic phases, this early peak is functionally relevant as it represents the critical time window for exosome-mediated delivery of regulatory miRNAs and proteins to the hippocampus. Such early entry allows ADSC-Exos to promptly suppress NLRP3 inflammasome activation and pyroptotic signaling triggered by kainic acid, thereby establishing the molecular basis for the delayed reduction in seizure burden and neuroinflammation observed during the chronic stage. We did not include a PKH-only sham control group in this study, previous reports have shown that PKH26-labeled MSC-derived exosomes administered systemically can reliably cross the blood–brain barrier and accumulate in hippocampal and cortical regions, which supports the reliability of our observations (38, 39). We chose the 2–6 h window for imaging based on previous studies indicating that MSC-derived exosomes tend to accumulate rapidly in the brain within this timeframe and remain detectable for 24–48 h (40, 41); while the models and labeling methods varied, the general distribution kinetics support this early-phase peak. Given the inherent limitations of PKH26 labeling, additional approaches such as co-immunostaining with exosomal markers could be considered in future work to provide more definitive evidence of exosome identity in brain tissue. These findings demonstrate rapid BBB penetration of ADSC-Exos post-injection, with their cerebral metabolism and duration of action potentially dynamically influenced by the local microenvironmental conditions. It should be noted that our current observations derive from experiments in healthy mice; neuroinflammatory states in epileptic models may significantly augment BBB permeability, and inter-study variability in kinetic profiles can arise from differences in experimental conditions and animal models (42, 43).

Moreover, we employed tail vein injection for exosome delivery, which is a simpler, less invasive approach with higher reproducibility and clinical translatability than intracranial administration, better aligning with the practical needs of epilepsy treatment. Although ADSC-Exos result in systemic distribution, they also exhibit inherent targeting capabilities. This is mediated by surface molecules such as CD47, which reduce phagocytic clearance, and integrins, which promote endothelial adhesion, thereby enabling efficient homing to brain pathology sites (44, 45). This intrinsic tropism, reflecting the homing properties of parental ADSCs (46, 47), facilitates selective accumulation in key epileptic regions like the hippocampus and cortex. Consistent with reports on other mesenchymal stem cell exosomes demonstrating therapeutic efficacy in brain injury (48), ischemia (49), and neurodegenerative models (20) upon intravenous injection. We administered ADSC-Exos weekly intravenously, following the established regimen of Wei Ying et al. (50), to optimize efficacy and minimize experimental risks. It should be emphasized that the current mechanistic interpretation of ADSC-Exos in regulating the TLE remains a predictive conclusion. Although the 7-day intravenous dosing regimen employed in this study mimics chronic clinical management strategies, the optimal dosing frequency for acute-phase intervention requires further investigation. Moreover, future studies should validate long-term safety in primate models and develop lyophilization techniques to improve exosome stability. It should also be noted that our study did not include a systematic dose–response or extended time-course evaluation of ADSC-Exos. The selected dose (100 µg per intravenous injection) was primarily based on commonly used ranges reported in previous studies (51, 52). While our biodistribution experiment confirmed brain entry with peak accumulation at 4 h, this does not substitute for a rigorous pharmacokinetic or pharmacodynamic assessment. Future studies will be required to compare different doses, establish minimal effective levels, and extend the time-course analyses to better guide translational application.

ADSC-Exos has demonstrated protective effects against acute seizures induced by various methods (28, 29). However, a key limitation of these studies lies in their experimental design: exosomes were consistently administered *before* seizure induction or epileptogenic injury. While this prophylactic approach confirms their neuroprotective potential, it poorly reflects real-world clinical scenarios—patients typically begin treatment only after diagnosis (53). To address this limitation, we employed the KA-induced mouse model - a classical acquired epilepsy model where kainic acid-induced status epilepticus (SE) serves as the epileptogenic insult, ultimately leading to recurrent spontaneous seizures following a 1–3 week latent period (54, 55).

Through staged post-modeling administration, we found that behavioral measures showed no significant intergroup differences during the acute SE phase (24 hours post-induction), suggesting that intense neuronal hyperexcitation and transient damage may initially overshadow exosome-mediated effects. However, the cumulative benefits of ADSC-Exos treatment progressively emerged during subsequent latent and chronic phases, demonstrating their time-dependent neuroprotective effects.

ADSC-Exos are enriched with diverse bioactive molecules (miRNAs, proteins, lipids) that collectively regulate gene expression and cellular signaling pathways, enabling multi-level interruption of the epileptogenic cascade. Specifically, ADSC-Exos deliver miRNAs such as miR-146a (56, 57), which suppress NLRP3 expression via NF-κB pathway inhibition, thereby limiting inflammasome assembly, Caspase-1 activation, and GSDMD cleavage (58, 59). This mechanism not only prevents pyroptotic cell death but also attenuates IL-1β release - a key cytokine exacerbating neuronal hyperexcitability and blood-brain barrier dysfunction in epilepsy (60). IL-1β and IL-18 are released as mature, circulating cytokines that provide a functional readout of inflammasome activation, and serum biomarkers are clinically accessible in patients, which improves the translational relevance of our measurements compared with invasive brain-tissue sampling. The observed IL-1β reduction in ADSC-Exos-treated groups confirms their anti-inflammatory reprogramming capacity. Furthermore, ADSC-Exos mitigate upstream pyroptosis triggers by stabilizing neuronal membranes to counteract KA-induced excitotoxicity (glutamate surge/calcium overload (61), while concurrently delivering antioxidant enzymes [e.g., superoxide dismutase (62)] and neurotrophic factors [e.g., brain-derived neurotrophic factor (63)] to alleviate oxidative stress and enhance neuronal survival. Critically, ADSC-Exos modulate microglial polarization through delivered miRNAs/proteins that shift phenotypes from pro-inflammatory M1 to anti-inflammatory M2 states (28, 64, 65), reducing IL-1β/GSDMD-N production and disrupting epileptogenic feedback loops. These integrated actions explain the reduced NLRP3 and Caspase-1 levels observed experimentally, demonstrating pyroptosis cascade disruption. Collectively, our findings indicate that the anti-epileptic effects of ADSC-Exos appear primarily mediated through NLRP3 inflammasome-associated pyroptosis pathway modulation, offering new translational avenues for temporal lobe epilepsy therapy.

Meanwhile, we employed bioinformatics to preliminarily explore the miRNA signatures of ADSC-Exos and epileptic hippocampi, aiming to identify potential molecular mechanisms by which ADSC-Exos might modulate NLRP3 inflammasome activation and contribute to anti-epileptic effects. Our bioinformatics analysis reveals that ADSC-Exos-derived miRNAs may exert anti-epileptic effects through a sophisticated, multi-layered regulatory network targeting the NLRP3 inflammasome-pyroptosis pathway. The identified miRNAs demonstrate systematic regulation across three functional tiers: they directly target core pyroptosis executor, extensively modulate upstream regulator, and regulate alternative inflammasome components. The most significantly regulated target was BRCC3 (targeted by 11 miRNAs), followed by PSTPIP1 (8 miRNAs), while miR-539-5p emerged as the most promiscuous regulator with predicted binding to 7 different pyroptosis-related genes. This network exhibits key therapeutic advantages including multi-target inhibition, synergistic effects through coordinated targeting of interconnected components, and pathway plasticity by modulating both canonical and alternative activation routes. Importantly, several of the candidate miRNAs have previously been implicated in neuroinflammation, neuronal excitability, or epilepsy models, which supports the biological plausibility of our predictions. While this approach provides useful hypotheses, it does not substitute for experimental validation. Moreover, our study did not perform direct molecular profiling (e.g., RNA-seq, proteomics, lipidomics) of the ADSC-Exos used here, and our conclusions about their cargo composition are therefore based on prior literature and external datasets. We did not perform qRT-PCR verification of candidate miRNAs in hippocampal tissue or exosomes across our experimental groups, and therefore the miRNA findings should be regarded as exploratory. Further experimental validation is essential to confirm these preliminary findings and support progress toward potential clinical applications.

Although our results emphasize the role of the NLRP3 inflammasome in mediating the beneficial effects of ADSC-Exos, it is important to recognize that exosomes contain diverse bioactive components that can act through multiple molecular targets. Potential parallel mechanisms may involve modulation of NF-κB signaling, oxidative stress, or ion channel regulation. Therefore, while our data highlight the association between ADSC-Exos and suppression of NLRP3-mediated pyroptosis, we cannot exclude the contribution of additional pathways. Further studies using pathway-specific inhibitors and omics-based profiling will be required to delineate the relative contributions of these mechanisms. In future work, comprehensive omics profiling (RNA-seq, proteomics, lipidomics) of the ADSC-Exos we isolated will be essential to validate and refine the mechanistic hypotheses proposed. Building on these data, high-priority miRNAs will be validated through qRT-PCR in ADSC-Exos and hippocampal tissue, followed by luciferase reporter assays to confirm direct binding to target genes. Functional relevance will be further tested using gain- and loss-of-function approaches (e.g., mimics, inhibitors, or viral vectors) both *in vitro* and *in vivo*, to determine whether modulation of these miRNAs alters NLRP3 inflammasome activity and seizure outcomes. Such integrated approaches will be crucial to translate the bioinformatic predictions into mechanistic insights and therapeutic strategies.

## 5 Conclusion

In this study, we demonstrated that tail vein administration of ADSC-Exos in KA-induced temporal lobe epilepsy mouse model effectively crosses the blood-brain barrier and targets the NLRP3 inflammasome-mediated pyroptosis pathway, significantly reducing seizure severity and frequency, attenuating neuroinflammation, and mitigating neuronal damage in the hippocampus, thereby exhibiting substantial therapeutic potential in alleviating TLE. Bioinformatics analysis revealed that these exosomal miRNAs coordinately suppress multiple nodes of the pyroptosis pathway. These findings provide a theoretical basis and experimental evidence for the application of ADSC-Exos in the treatment of temporal lobe epilepsy and other neurological disorders. 

## Data Availability

The original contributions presented in the study are included in the article/**Supplementary Material**. Further inquiries can be directed to the corresponding authors.
