# Supplementary material for: Adipose-derived stem cell exosomes suppress NLRP3-mediated neuronal pyroptosis to attenuate seizures in a kainic acid-induced temporal lobe epilepsy model
Source: Front Immunol. 2025 Oct 30;16:1691814. doi: 10.3389/fimmu.2025.1691814 (PMC12611705; doi:10.3389/fimmu.2025.1691814)
Supplement: Supplementary file 1 [file Table1.docx]

Primer Information:

| **Gene Name** | **Direction** | **Primer Sequence (5‘→3’)** |
| --- | --- | --- |
| GSDMD | Forward | CCATCGGCCTTTGAGAAAGTG |
|  | Reverse | ACACATGAATAACGGGGTTTCC |
| NLRP3 | Forward | TGGATGGGTTTGCTGGGAT |
|  | Reverse | CTGCGTGTAGCGACTGTTGAG |
| Caspase-1 | Forward | TTGAAAGACAAGCCCAAGGTG |
|  | Reverse | CTGGTGTTGAAGAGCAGAAAGC |
| IL-1β | Forward | ACCTTCCAGGATGAGGACATGA |
|  | Reverse | AACGTCACACACCAGCAGGTTA |
| β-actin | Forward | CACGATGGAGGGGCCGGACTCATC |
|  | Reverse | TAAAGACCTCTATGCCAACACAGT |
